# Dexmedetomidine Inhibits Inflammatory Reaction in Lung Tissues of Septic Rats by Suppressing TLR4/NF-**κ**B Pathway

**DOI:** 10.1155/2013/562154

**Published:** 2013-04-08

**Authors:** Yuqing Wu, Yingchun Liu, He Huang, Yangzi Zhu, Yong Zhang, Fuzhao Lu, Ce Zhou, Li Huang, Xin Li, Chenghua Zhou

**Affiliations:** ^1^Jiangsu Province Key Laboratory of Anesthesiology, Xuzhou Medical College, Tongshan Road 209, Xuzhou 221004, China; ^2^Department of Anesthetic Pharmacology, Xuzhou Medical College, Tongshan Road 209, Xuzhou 221004, China; ^3^The Sixth People's Hospital of Xuzhou City, Huaihai Road, Xuzhou 221002, China; ^4^Department of Pharmacology, School of Pharmacy, Xuzhou Medical College, Tongshan Road 209, Xuzhou 221004, China

## Abstract

Dexmedetomidine has been reported to reduce mortality in septic rats. This study was designed to investigate the effects of dexmedetomidine on inflammatory reaction in lung tissues of septic rats induced by CLP. After induction of sepsis, the rats were treated with normal saline or dexmedetomidine (5, 10, or 20 **μ**g/kg). The survival rate of septic rats in 24 h was recorded. The inflammation of lung tissues was evaluated by HE stain. The concentrations of IL-6 and TNF-**α** in BALF and plasma were measured by ELISA. The expressions of TLR4 and MyD88 were measured by western blotting. The activation of NF-**κ**B in rat lung tissues was assessed by western blotting and immunohistochemistry. It was found that the mortality rate and pulmonary inflammation were significantly increased in septic rats. IL-6 and TNF-**α** levels in BALF and plasma, NF-**κ**B activity, and TLR4/MyD88 expression in rat lung tissues were markedly enhanced after CLP. Dexmedetomidine (10 and 20 **μ**g/kg) significantly decreased mortality and pulmonary inflammation of septic rats, as well as suppressed CLP-induced elevation of TNF-**α** and IL-6 and inhibited TLR4/MyD88 expression and NF-**κ**B activation. These results suggest that dexmedetomidine may decrease mortality and inhibit inflammatory reaction in lung tissues of septic rats by suppressing TLR4/MyD88/NF-**κ**B pathway.

## 1. Introduction

Sepsis is a main problem in the intensive care unit (ICU) and carries a very high mortality rate. It involves a network of proinflammatory cytokines such as TNF-*α* and IL-6 which are overexpressed after various noxious insults, especially bacterial infections [1–4]. Enhanced inflammatory reaction in lung tissues is believed to be an important component of the pathophysiology of sepsis [5]. Patients with endotoxemia are under the hyperstress state and often require drugs for sedation and analgesia to reduce anxiety and stress. 

Dexmedetomidine, a selective agonist of *α*_2_-adrenergic receptor, is a potent sedative agent for critically ill patients and also provides effective analgesia [6]. The site of action for dexmedetomidine's sedative effects is thought to be the locus ceruleus in the midbrain, where it inhibits the release of neurotransmitters from synaptic terminals, thus inhibiting neuronal signaling and decreasing wakefulness. Dexmedetomidine's analgesic effects are mediated in the dorsal horn of spinal cord, where it is thought to decrease the firing of ascending nociceptive neurons. These actions result in effective sedation and analgesia without respiratory depression and drug-dependency issues. Along with its beneficial effects, dexmedetomidine was reported to exert potential anti-inflammatory effect during endotoxemia. Previous study revealed that dexmedetomidine, a new sedative agent, significantly reduced mortality and decreased the levels of inflammatory cytokines during endotoxemia in rats [7]. However, the detailed mechanisms by which dexmedetomidine regulates inflammatory responses during endotoxemia have not been clearly revealed.

Toll-like receptors (TLRs) are a family of transmembrane proteins and act as signal transduction molecules. Toll-like receptor 4 (TLR4) has been considered the main sensor to recognize the pathogen-associated molecular patterns (PAMPs). TLR4 is a member of the IL-1R/TLR superfamily that is required for LPS responsiveness and is involved in the host defense against Gram-negative bacteria [8]. Stimulation of TLR4 can activate NF-kappaB protein through myeloid differentiation factor 88 (MyD88) dependent or independent pathway. After ligand binding, TLRs/IL-1Rs dimerize and undergo the conformational change required for the recruitment of downstream signaling molecules and stimulation of downstream kinases, including activation of the transcriptional factors NF-*κ*B [9]. The activation of NF-*κ*B leads to the induction of genes encoding proinflammatory cytokines such as IL-6 and TNF-*α* [10].

This study aims to investigate the effects of dexmedetomidine on inflammatory reaction in lung tissues of septic rats induced by cecal ligation and puncture (CLP) and relevant mechanisms. We hypothesized that dexmedetomidine could suppress inflammatory response in rat lung tissues during endotoxemia induced by CLP. Furthermore, we probed whether dexmedetomidine could attenuate CLP-induced pulmonary inflammation through inhibiting TLR4/MyD88 expression and NF-*κ*B activation, reducing proinflammatory cytokines production in septic rats.

## 2. Materials and Methods

### 2.1. Animals

Male Sprague-Dawley rats (250–300 g) were obtained from Shanghai Animal Center (Shanghai, China). The rats were housed at 23°C with 12 h of light and 12 h of darkness each day and allowed free access to food and water. All experiments were performed in accordance with the institutional criteria for the care and use of laboratory animals in research.

### 2.2. Cecal Ligation and Puncture (CLP) Operation

The rats were anesthetized with an intraperitoneal injection of pentobarbital sodium 50 mg/kg. Polymicrobial sepsis was induced by CLP as previously described [11]. The procedure was performed under sterile conditions with the abdominal skin disinfected with 75% alcohol. Laparotomy was conducted through 2 cm lower-midline incision. The cecum was exposed and ligated immediately distal to the ileocecal valve to avoid intestinal obstruction and then punctured twice with an 18-gauge needle, squeezed gently to force out a small amount of feces, and then returned to the abdominal cavity. The abdomen is closed with 3–0 silk sutures in two layers. 

### 2.3. Experimental Protocol

Rats were randomly divided into five groups: sham operation, CLP, small dose, medium dose, and large dose of dexmedetomidine. Following completion of CLP or sham operation dexmedetomidine or normal saline were administered. The rats in sham operation and CLP groups were administered by intraperitoneal injection normal saline, and the rats in small, medium, and large dose dexmedetomidine groups were administered by intraperitoneal injection 5, 10, and 20 *μ*g/kg dexmedetomidine, respectively. Dexmedetomidine or normal saline was administered by intraperitoneal injection at 0, 2, 4, and 6 h after operation.

### 2.4. Mortality Rate

Forty rats were randomly divided into five groups (8 per group) as aforementioned. The animals were performed by CLP or sham operation and administered as previously stated. All the animals were monitored after operation and administration. The time when animal died was recorded. The animals were observed up to 24 hours after operation and sacrificed by lethal sodium pentobarbital injection. The mortality rate within 24 hours was calculated.

### 2.5. Plasma, Bronchoalveolar Lavage Fluid, and Lung Tissues Collection

Venous blood samples were drawn at 0, 2, 4, and 6 hours after operation for the measurement of plasma cytokines. At 8 hours after operation or as soon as the animal died, the left main bronchus was tied and the left lung was removed. The upper lobe of the left lung tissues was snap-frozen in liquid nitrogen and stored at −80°C for subsequent protein detection by western blot, and the lower lobe of the left lung was used to HE stain and immunohistochemistry. The right lung from each group was lavaged with sterile normal saline through the tracheostomy tube as previous report [12], and then bronchoalveolar lavage fluid (BALF) was collected for measurement of cytokines by enzyme-linked immunosorbent assay (ELISA). 

### 2.6. HE Stain for Lung Tissues

Formaldehyde-fixed lower lobe of the left lung was embedded in paraffin wax, serial sectioned, and stained with hematoxylin and eosin. Histologic changes including alveolar wall edema, congestion, hemorrhage, and inflammatory cells infiltration were evaluated under a light microscope to assess pulmonary inflammation according to the previous report [12]. Each histologic characteristic was scored on a scale of 0 (normal) to 5 (severe) by a pathologist who was blind to this study. The overall pulmonary inflammation was categorized according to the sum of the score (0–5: normal to minimal inflammation; 6–10: mild inflammation; 11–15: moderate inflammation; 16–20: severe inflammation).

### 2.7. ELISA for IL-6 and TNF-*α*

The levels of IL-6 and TNF-*α* in plasma and BALF were detected by ELISA. IL-6 and TNF-*α* were measured using commercially available ELISA kits according to the protocols of the kits. Briefly, cell free supernatant was added to each well of a monoclonal rabbit anti-rat IL-6 or TNF-*α* antibody coated microtitre plates (ELISA plates) for 12 h at 4°C. Unbound material was washed off and a biotinylated monoclonal rabbit anti-rat IL-6 or TNF-*α* antibody was used for 45 min. Bound antibody was detected by addition of avidin-peroxidase for 30 min followed by incubation of the ABTS substrate solution. Absorbance was measured 20 min after addition of substrate. A standard curve was constructed using various dilutions of IL-6 or TNF-*α* standard preparation. The levels of IL-6 and TNF-*α* were determined by extrapolation of absorbance to the standard curve. 

### 2.8. Western Blot for TLR4, MyD88, and NF-*κ*B

Frozen rat lung tissues were homogenized and the lysates were prepared in ice-cold lysis buffer. Nuclear extracts were collected and stored at −80°C for western blot analyses of nuclear translocation of NF-*κ*B p65 protein. The extracts were normalized for equal amounts of total protein measured by the bicinchoninic acid (BCA) method. Seventy micrograms protein from each sample was separated on a sodium dodecyl sulfate-polyacrylamide gel and transferred to PVDF membranes. The membranes were blocked with 5% nonfat milk and incubated overnight with primary anti-NF-*κ*B p-65 antibody (Cell Signaling Technology, USA), anti-TLR4 antibody (Santa Cruz), or anti-MyD88 antibody (Santa Cruz) at 4°C, followed by incubation with the suitable HRP-conjugated secondary antibody for 4 hours. Cellular GAPDH protein was immunodetected as the internal standard. 

### 2.9. Immunohistochemistry for NF-*κ*B

NF-*κ*B p-65 protein activity was also observed by immunohistochemistry technique. Briefly, sections were microwaved for antigen retrieval and pretreated with 0.3%  H_2_O_2_. Subsequently, the sections were blocked with goat serum and incubated in a primary antibody solution containing rabbit anti-NF-*κ*B p-65 antibody overnight at 4°C. After washing, the samples were incubated in a suitable secondary antibody solution for 2 h at room temperature. Finally, the sections were incubated in HRP-streptavidin (1 : 100, Zhongshan Biotechnology) for 1 h at room temperature, and the color reaction was developed with diaminobenzidine (DAB). The sections were counterstained, dehydrated, coverslipped, and analyzed under light microscope. Five high-power fields per slide were observed. The degree of NF-*κ*B activation was expressed as percentage of nuclear NF-*κ*B p-65 positive cells to total alveolar epithelial cells.

### 2.10. Statistical Analysis

Data were presented as mean ± SD. SPSS-13.0 software was used for data analysis. Differences among groups were determined by one-way analysis of variance (ANOVA), followed by a post hoc test (Bonferroni's method). The mortality rates among groups were compared using the Kaplan Meier methods. Statistical significance was defined as *P* < 0.05. 

## 3. Results

### 3.1. Mortality Rate

**Figure 1:**
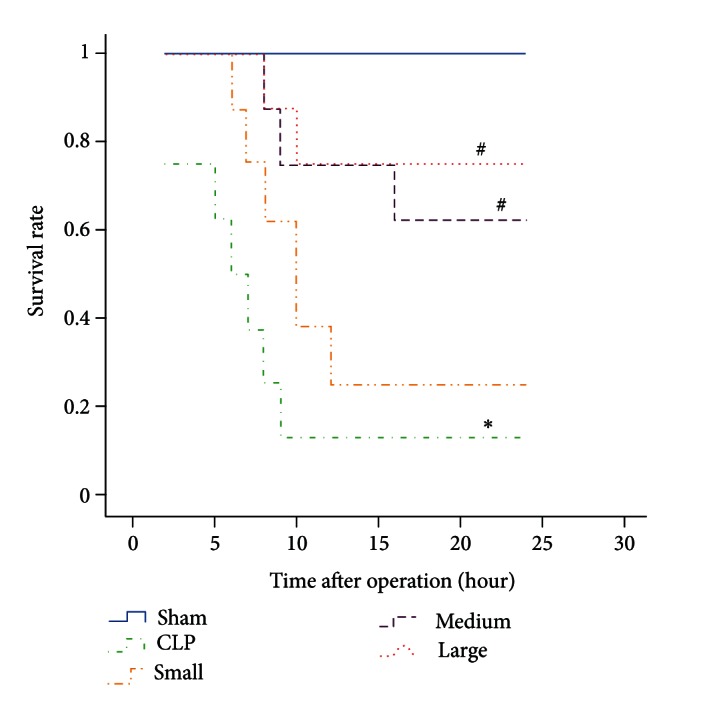
Effect of dexmedetomidine on survival curves of septic rats induced by CLP. Sham: sham operation group; CLP: cecal ligation and puncture operation group; Small: 5 *μ*g/kg dexmedetomidine treatment group; Medium: 10 *μ*g/kg dexmedetomidine treatment group; Large: 20 *μ*g/kg dexmedetomidine treatment group. *n* = 8. The survival rate at 24 h after operation was analyzed. *P < 0.01, versus sham group; ^#^ *P* < 0.01, versus CLP group.

As shown in Figure 1, the mortality rates in 24 hours after operation were 0%, 87.5%, 75%, 37.5%, and 25%, respectively for sham, CLP, small, medium and large dose groups. Compared to sham group, the mortality rate in CLP group markedly increased. However, medium and large doses of dexmedetomidine significantly suppressed the elevated mortality rate induced by CLP. There is no significant difference between small dose dexmedetomidine group and CLP group for mortality rate.

### 3.2. Histopathological Observation in Lung Tissues

**Figure 2:**
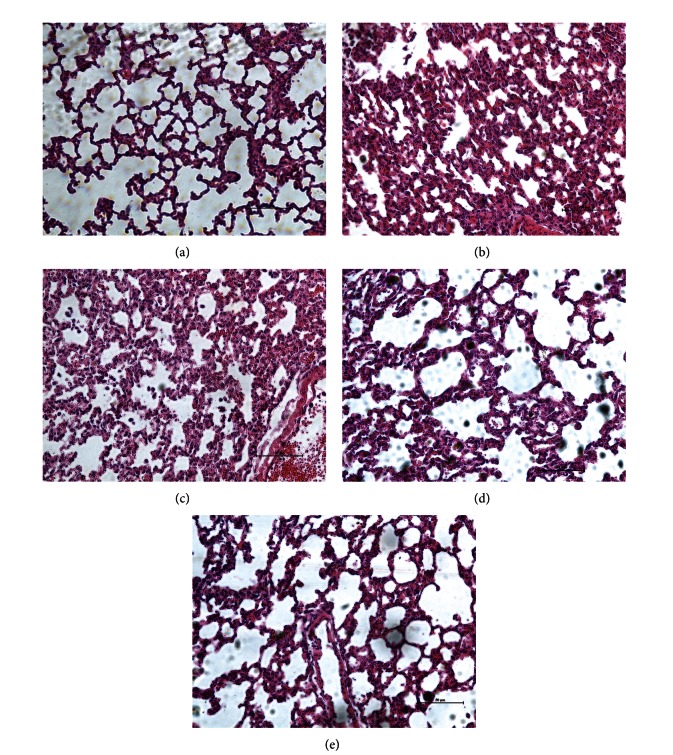
Microscopic findings of the lung tissues stained with hematoxylin and eosin. (a) Sham operation group. The lung tissues showed normal to minimal pulmonary inflammation. (b) CLP group. The lung tissues showed severe pulmonary inflammation. (c) Small dose (5 *μ*g/kg) group. The lung tissues showed severe pulmonary inflammation. (d) Medium dose (10 *μ*g/kg) group. The lung tissues showed moderate pulmonary inflammation. (e) Large dose (20 *μ*g/kg) group. The lung tissues showed mild pulmonary inflammation. (magnification 200x).

**Figure 3:**
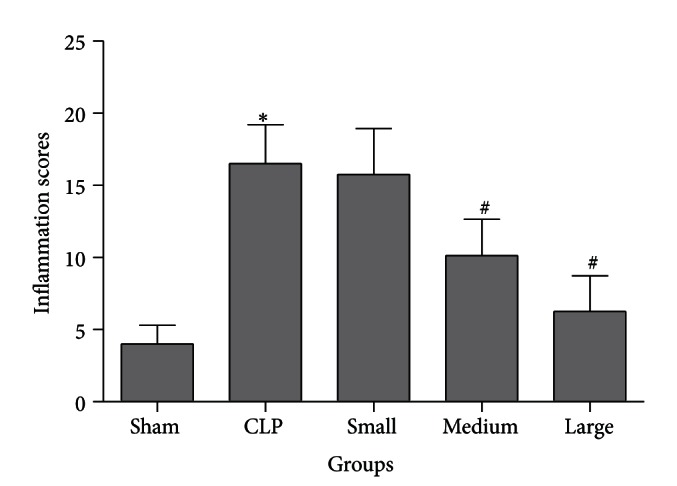
Pulmonary inflammation scores of rats from different groups. Sham: sham operation group; CLP: cecal ligation and puncture operation group; Small: 5 *μ*g/kg dexmedetomidine treatment group; Medium: 10 *μ*g/kg dexmedetomidine treatment group; Large: 20 *μ*g/kg dexmedetomidine treatment group. Data are expressed as mean ± SD, *n* = 8. *P < 0.01, versus sham group; ^#^ *P* < 0.01, versus CLP group.

The representative micrographs in Figure 2 represent eight samples in each group. Observed under light microscope by hematoxylin and eosin staining, rat lung tissue sections in sham group showed normal alveolar architecture (Figure 2(a)). Rats in CLP group exhibited marked lung histopathologic abnormalities, characterized by alveolar wall edema, congestion, leakage of microvessel, and inflammatory cells infiltration (Figure 2(b)). Small dose of dexmedetomidine failed to improve the histopathologic abnormalities. However, after treatment with medium and large doses of dexmedetomidine, lung tissues appeared relatively better, with a prominent decrease in inflammation response (Figures 2(d) and 2(e)) compared to CLP group. Findings of the pulmonary inflammation score paralleled the findings of the histopathological observation (Figure 3).

### 3.3. Levels of IL-6 and TNF-*α* in Plasma and BALF

**Figure 4:**
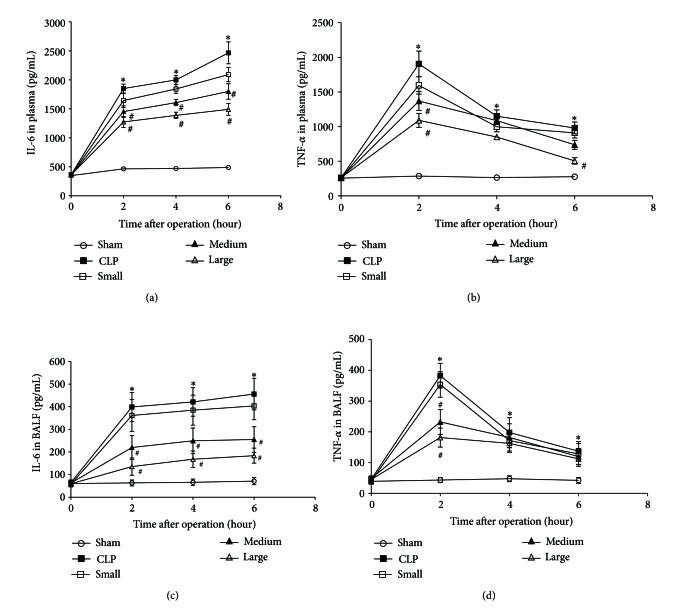
Levels of interleukin-6 (IL-6) and tumor necrosis factor-*α* (TNF-*α*) in plasma (*n* = 6) and bronchoalveolar lavage fluid (BALF) (*n* = 8). (a) IL-6 in plasma. (b) TNF-*α* in plasma. (c) IL-6 in BALF. (d) TNF-*α* in BALF. Data are expressed as mean ± SD. *P < 0.01, versus sham group; ^#^ *P* < 0.01, versus CLP group.

As shown in Figure 4, the baseline values of IL-6 and TNF-*α* in the five groups were similar. However, both IL-6 and TNF-*α* levels in plasma and BALF of rats were markedly increased at 2, 4, and 6 hours after CLP operation compared to those in sham group. Medium and large doses of dexmedetomidine significantly inhibited the production of IL-6 and TNF-*α* in plasma and BALF induced by CLP. While small dose of dexmedetomidine did not obviously affect the levels of these proinflammatory cytokines in plasma and BALF of CLP rats. 

### 3.4. Western Blot Analysis for TLR4/MyD88 Expression in Lung

**Figure 5:**
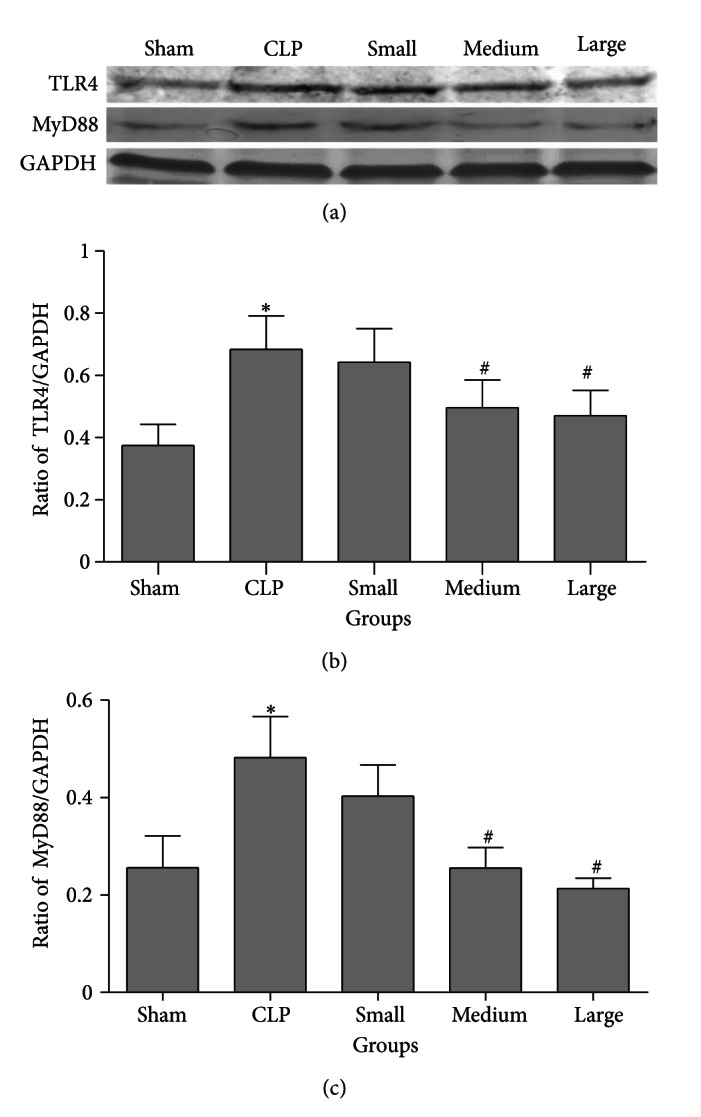
Effect of dexmedetomidine on TLR4 and MyD88 expression in lung tissues of septic rats by western blotting. Sham: sham operation group; CLP: cecal ligation and puncture operation group; Small: 5 *μ*g/kg dexmedetomidine treatment group; Medium: 10 *μ*g/kg dexmedetomidine treatment group; Large: 20 *μ*g/kg dexmedetomidine treatment group. GAPDH: glyceraldehyde phosphate dehydrogenase. Data are expressed as mean ± SD, *n* = 8. *P < 0.01, versus sham group; ^#^ *P* < 0.01, versus CLP group.

In order to clarify the mechanism through which dexmedetomidine inhibits pulmonary inflammation of septic rats induced by CLP, we further investigated the expression of TLR4/MyD88 in lung tissues. In this study, TLR4/MyD88 expression in lung tissues of rats from CLP group was markedly upregulated compared to that in sham group. Medium and large doses of dexmedetomidine significantly suppressed TLR4/MyD88 expression in lung of septic rats. However, small dose of dexmedetomidine did not markedly reduce CLP induced overexpression of TLR4/MyD88 (Figure 5).

### 3.5. Western Blot Analysis for NF-*κ*B Activity

**Figure 6:**
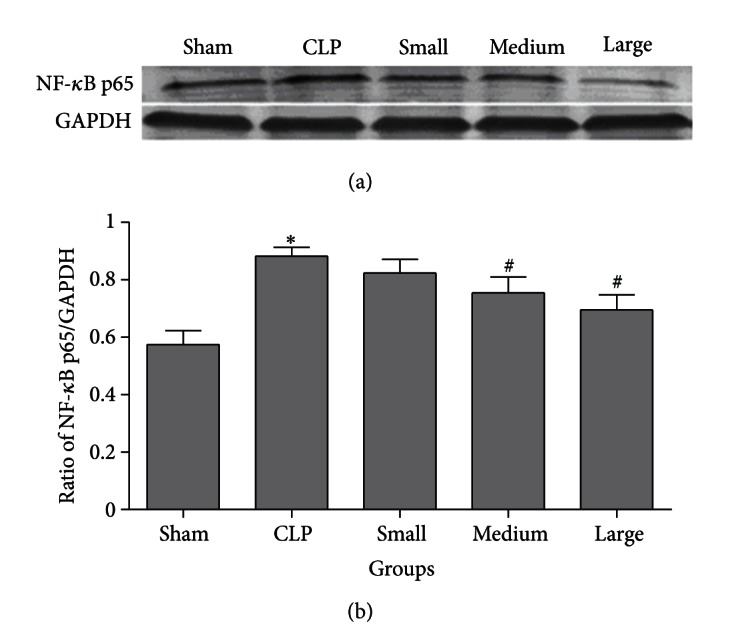
Effect of dexmedetomidine on NF-*κ*B p-65 activity in lung nuclear extracts of septic rats by western blotting. Sham: sham operation group; CLP: cecal ligation and puncture operation group; Small: 5 *μ*g/kg dexmedetomidine treatment group; Medium: 10 *μ*g/kg dexmedetomidine treatment group; Large: 20 *μ*g/kg dexmedetomidine treatment group. GAPDH: glyceraldehyde phosphate dehydrogenase. Data are expressed as mean ± SD, *n* = 8. *P < 0.01, versus sham group; ^#^ *P* < 0.01, versus CLP group.

As shown in Figure 6, the expression of NF-*κ*B p65 in nuclear extracts was markedly upregulated after CLP operation compared to that in sham group. Dexmedetomidine at the medium and large doses significantly attenuated activation of NF-*κ*B p65 in septic rat lung. However, small dose of dexmedetomidine did not obviously inhibit the activation of NF-*κ*B p65 induced by CLP operation. 

### 3.6. Immunohistochemistry for NF-*κ*B Activity

**Figure 7:**
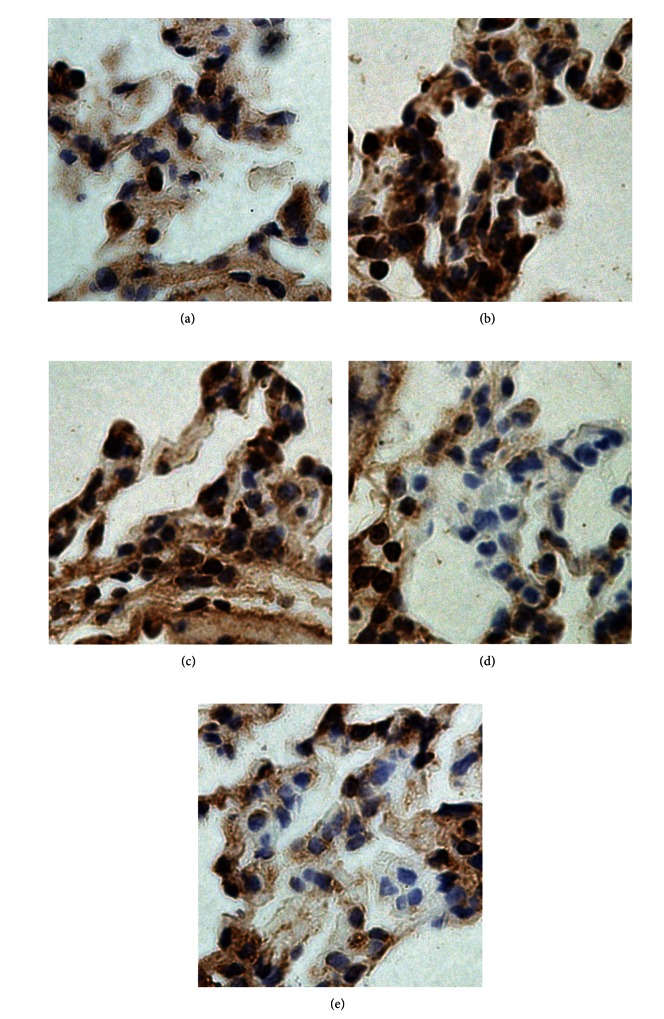
Effect of dexmedetomidine on NF-*κ*B p-65 activation in lung tissues of septic rats by immunohistochemistry staining. (a) Sham operation group; (b) cecal ligation and puncture operation group; (c) 5 *μ*g/kg dexmedetomidine treatment group; (d) 10 *μ*g/kg dexmedetomidine treatment group; (e) 20 *μ*g/kg dexmedetomidine treatment group (magnification 400x).

**Figure 8:**
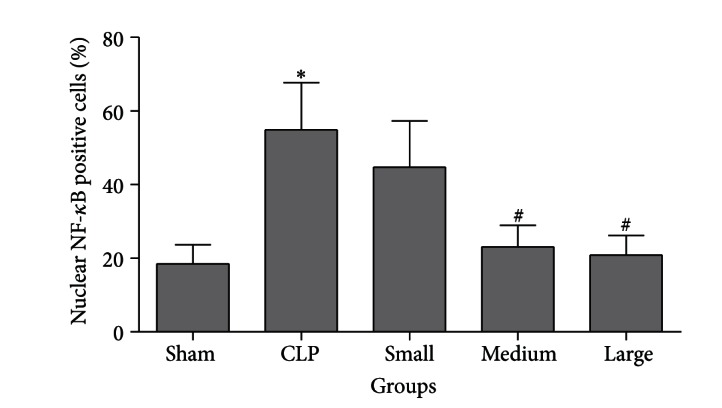
Effect of dexmedetomidine on NF-*κ*B p-65 activation in lung tissues of septic rats by immunohistochemistry staining. Sham: sham operation group; CLP: cecal ligation and puncture operation group; Small: 5 *μ*g/kg dexmedetomidine treatment group; Medium: 10 *μ*g/kg dexmedetomidine treatment group; Large: 20 *μ*g/kg dexmedetomidine treatment group. The degree of NF-*κ*B activation was indicated as percentage of nuclear NF-*κ*B p-65 positive cells to total alveolar epithelial cells. Data are expressed as mean ± SD, *n* = 8. *P < 0.01, versus sham group; ^#^ *P* < 0.01, versus CLP group.

The nuclear positive staining represents the activated form of NF-kappa B. In CLP group, about 54% of alveolar epithelial cells expressed nuclear positive staining, as compared to about 18.5% in sham group (*P* < 0.01). The percentage of cells showing nuclear positive staining significantly decreased after treatment with dexmedetomidine at medium and high doses compared to CLP group (23.1 ± 5.8%, *P* < 0.01; 20.9 ± 5.3%, *P* < 0.01; versus 54.9 ± 12.8%, Figures 7 and 8). 

## 4. Discussion

In the present study, we investigated the effect of dexmedetomidine on mortality and inflammatory responses in lung tissues of septic rats induced by CLP. It was found that dexmedetomidine at the doses of 10 and 20 *μ*g/kg decreased CLP-induced pulmonary inflammation and mortality, reduced production of IL-6 and TNF-*α* in plasma and BALF of septic rats, and suppressed TLR4/MyD88 expression and NF-*κ*B activation in lung of endotoxemia rats induced by CLP. Our results suggest that dexmedetomidine may modulate some of the inflammatory responses in lung of septic rats in vivo by suppressing TLR4/MyD88/NF-*κ*B pathway, which may contribute to decrease the mortality of septic rats induced by CLP. 

Acute lung injury characterized by overinflammatory reaction is a serious consequence of sepsis [5]. Pulmonary inflammatory response, including upregulation of various proinflammatory factors and infiltration of many inflammatory cells, is crucial in endotoxemia and is also one of the important reasons leading to high mortality. Therefore, attenuating excessive pulmonary inflammation during endotoxemia is beneficial to decrease the mortality rate of septic patients. Previous study has demonstrated that dexmedetomidine reduced mortality rate and had an inhibitory effect on inflammatory response during endotoxemia induced by intravenous injection of Escherichia coli endotoxin [7]. Our results that dexmedetomidine markedly decreased CLP-induced mortality, reduced production of IL-6 and TNF-*α* in plasma, and attenuated inflammatory histopathological changes in lung tissues of septic rats induced by CLP were in concert with the previous report [7]. In addition, several studies [13–15] have demonstrated that dexmedetomidine could exert a potential protective effect by suppressing inflammatory responses on ventilator, lipopolysaccharide, or *α*-naphthylthiourea-induced acute lung injury. Although the present and previous studies have shown the regulatory effects of dexmedetomidine on inflammatory reactions in acute lung injury, the exact mechanisms responsible for these actions are not well understood. 

Toll-like receptor 4 (TLR4) is a transmembrane receptor protein with extracellular leucine-rich repeated domains and a cytoplasmic signaling domain. TLR4 is involved in immune responses, especially in the activation of innate immunity against foreign pathogens and microorganisms, but it also triggers adaptative immunity [16–18]. TLR4 is a CD-14 associated transmembrane signal transducer, which is necessary for the LPS-induced cellular response [19–21]. Recent studies have suggested that the association of TLR4 with myeloid differentiation factor 88 (MyD88) may induce the activation of IL-1R associated kinase and TNF receptor-associated factor [22], which triggers inflammatory cascade reactions. 

Nuclear factor kappa B (NF-*κ*B) is an important nuclear transcription factor. NF-*κ*B heterodimer consists of p50 and p65 (Rel A) subunits. It plays a pivotal role in immune and inflammatory responses through the regulation of the expression of several proteins, including proinflammatory cytokines, chemokines, and adhesion molecules. Uncontrolled activation of the NF-*κ*B pathway is involved in the pathogenesis of many acute and chronic inflammatory diseases. In its inactive state, the NF-kappaB dimer is present in the cytosol, where it is bound to an inhibitory protein, I-kappaB. Activation of NF-kappaB by several stimuli induces the release and degradation of the inhibitory protein I-kappaB from the dimeric complex [23], followed by phosphorylation of NF-kappaB p65 and translocation to the nucleus [24, 25]. 

TLR4-mediated signaling pathways mainly stimulate the activation of NF-*κ*B. In the nucleus, NF-kappaB is bound to corresponding sites to regulate transcription of many proinflammatory genes such as IL-6 and TNF-*α*. In the present study, polymicrobial sepsis animal model was established by CLP. The results showed that both TLR4/MyD88 expression and NF-*κ*B activation were significantly upregulated in lung tissues of septic rats. Here, we also showed that dexmedetomidine (10 and 20 *μ*g/kg) treatment could reduce CLP-induced enhanced TLR4/MyD88 expression and NF-*κ*B activation in rat lung. Since TLR4 is an essential upstream sensor for LPS from pathogens and microorganisms and may mediate the NF-*κ*B activation through MyD88 dependent pathway, and NF-*κ*B activation can increase the production of IL-6 and TNF-*α*, it is possible that dexmedetomidine reduces the production of these cytokines and attenuates pulmonary inflammation by suppressing TLR4/MyD88/NF-*κ*B signaling pathway in lung of polymicrobial septic rats induced by CLP. Our results suggested that suppression of TLR4/MyD88/NF-*κ*B signaling may be the probable mechanism through which dexmedetomidine attenuated inflammatory responses in lung of septic rats induced by CLP. In addition, small dose of dexmedetomidine did not affect the expression of TLR4/MyD88 and the activation of NF-*κ*B in lung tissues of septic rats. These results were consistent with the effect that small dose of dexmedetomidine failed to inhibit pulmonary inflammation and decrease mortality rate of septic rats induced by CLP.

In conclusion, the present findings indicated that dexmedetomidine inhibited pulmonary inflammation and reduced the production of proinflammatory cytokines IL-6 and TNF-*α* by attenuation of TLR4/MyD88/NF-*κ*B pathway activation in septic rats induced by CLP. These properties of dexmedetomidine may contribute to decrease the mortality rate of septic rats.
